# Mechanisms and benefits of cardiac rehabilitation in individuals with stroke: emerging role of its impact on improving cardiovascular and neurovascular health

**DOI:** 10.3389/fcvm.2024.1376616

**Published:** 2024-05-02

**Authors:** Sara J. Cuccurullo, Talya K. Fleming, Hayk Petrosyan, Daniel F. Hanley, Preeti Raghavan

**Affiliations:** ^1^ Department of Physical Medicine and Rehabilitation, JFK Johnson Rehabilitation Institute at Hackensack Meridian Health, Edison, NJ, United States; ^2^ Brain Injury Outcomes, Johns Hopkins Medical Institutions, Baltimore, MD, United States; ^3^ Department of Physical Medicine and Rehabilitation and Neurology, Johns Hopkins University School of Medicine, Baltimore, MD, United States

**Keywords:** stroke, cerebrovascular accident, stroke rehabilitation, cardiac rehabilitation, exercise, physical activity, neurorehabilitation, stroke recovery

## Abstract

Human and animal studies have demonstrated the mechanisms and benefits of aerobic exercise for both cardiovascular and neurovascular health. Aerobic exercise induces neuroplasticity and neurophysiologic reorganization of brain networks, improves cerebral blood flow, and increases whole-body VO2_peak_ (peak oxygen consumption). The effectiveness of a structured cardiac rehabilitation (CR) program is well established and a vital part of the continuum of care for people with cardiovascular disease. Individuals post stroke exhibit decreased cardiovascular capacity which impacts their neurologic recovery and extends disability. Stroke survivors share the same risk factors as patients with cardiac disease and can therefore benefit significantly from a comprehensive CR program in addition to neurorehabilitation to address their cardiovascular health. The inclusion of individuals with stroke into a CR program, with appropriate adaptations, can significantly improve their cardiovascular health, promote functional recovery, and reduce future cardiovascular and cerebrovascular events thereby reducing the economic burden of stroke.

## Introduction

Vascular disease impacts not only the cardiovascular system but also the cerebrovascular system with ischemic heart disease and stroke being the top-ranked causes of disability (1). Individuals with stroke have significant atherosclerotic complications within their vascular system and a high prevalence of cardiovascular disease (2–4). Approximately 75% of stroke patients exhibit cardiovascular comorbidities, and the prevalence of coronary artery disease among stroke survivors is estimated to range from 32% to 65% (5, 6). Stroke leaves approximately one-third of survivors dependent, straining healthcare systems (7). The burden of stroke is especially high in individuals with cardiac disease (8), and treatment of stroke necessitates the modification of risk factors for cardiac disease, including changes in physical activity levels (2, 9). Physical activity is body movement that is produced by the contraction of skeletal muscle that substantially increases energy expenditure, whereas exercise is a type of physical activity that involves planned, structured bodily movement done to maintain or improve physical fitness (10). More specifically, aerobic exercise is a type of exercise that involves large muscle groups, is rhythmic and repetitive in nature, can be maintained continuously, and increases heart rate and oxygen consumption (11). Aerobic exercise has been shown to have many benefits in improving cardiovascular health in individuals with stroke (12–14). Hence, the American Heart Association (AHA) and American Stroke Association (ASA) guidelines recommend moderate-intensity aerobic exercise, lasting 20–60 min, performed 3–5 times a week for stroke survivors (15–17). However, a major challenge is that widespread implementation of these guidelines have proven very difficult (18–21). Studies have shown that individuals with stroke spend approximately 80% of their time in sedentary behaviors (22, 23). As a result of a significant reduction in their normal physical activity, stroke survivors are in fact more likely to experience the negative effects of deconditioning, including the increased risk of cardiovascular events and decreased cardiovascular health (24, 25). In the first 90 days post-stroke, 19% of survivors of stroke experience at least one serious cardiac adverse event, and cardiac mortality is the second leading cause of death during this critical time (26–28).

The recommendations for physical activity for secondary stroke prevention in individuals with deficits after stroke include supervision by a cardiac rehabilitation professional in addition to routine rehabilitation (29). Programs that use theoretical models of behavior change, proven techniques, and multidisciplinary support are needed to ensure that individuals with stroke receive the physical activity interventions required. Cardiac rehabilitation is a comprehensive, structured program that provides prescribed aerobic exercise in addition to medical evaluation, cardiac risk factor modification, education, and counseling (30). Traditional cardiac rehabilitation (CR) meets the recommended requirements by the AHA/ASA and if properly adapted can be implemented even in individuals with movement deficits after a stroke to improve cardiovascular health (31–35). However, more investigation is needed to determine the optimal intensity, timing, and long-term benefits of CR post-stroke. Importantly, CR provides a structure for physical activity in a motivational environment and risk factor education that will provide individuals recovering from stroke with a comprehensive program and opportunities to change risk factor behaviors similar to individuals with cardiac disease. This review synthesizes the available literature regarding the benefits and mechanisms of how CR, and its major component, aerobic exercise, affects both cardiovascular and neurovascular health.

### Mechanisms of aerobic exercise and its impact on cardiovascular and neurovascular health

**Figure 1:**
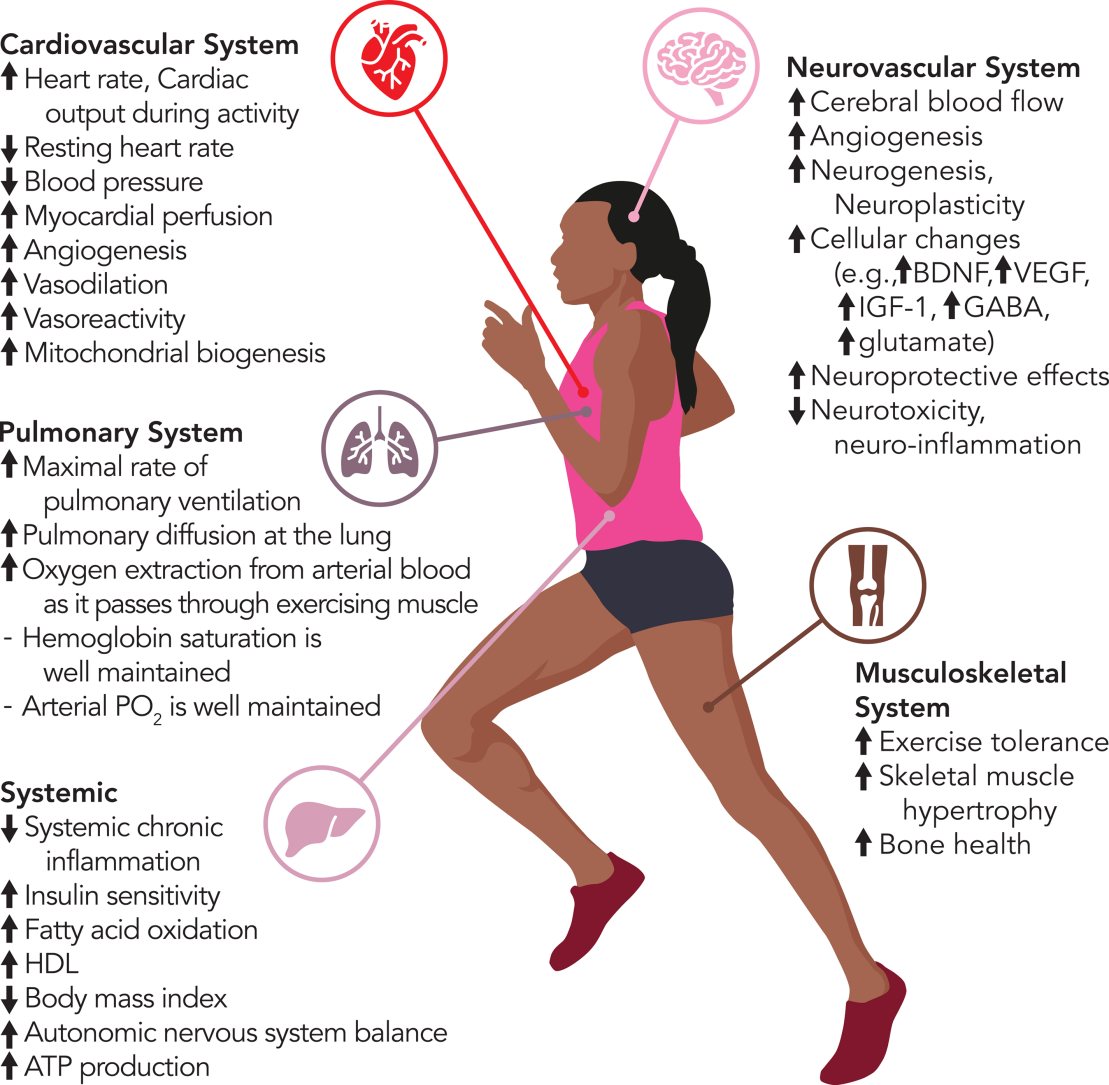
Aerobic exercise-induced changes in body systems.

Aerobic exercise challenges homeostasis and mediates substantial changes across the cardiovascular, pulmonary, musculoskeletal, neurovascular and metabolic systems, which occur in cells, tissues, and organs in direct response to the increased metabolic demand on the body (Figure 1) (36). The main mechanisms through which aerobic exercise impacts cardiovascular health include improved oxygen delivery, changes in vasculature and peripheral tissues, regulation of inflammation, and promotion of vasodilation and angiogenesis. Exercise upregulates the expression of hypoxia-inducible factor (HIF)1α and peroxisome proliferator-activated receptor γ co-activator 1α (PGC1α), which leads to the production of vascular endothelial growth factor (VEGF)—a critical factor for angiogenesis (37–39). Moreover, physical exercise increases mitochondrial biogenesis in skeletal muscle, myotubes, and cardiomyocytes (40, 41). Additionally, several studies have presented evidence indicating that exercise training induces changes in mitochondrial reactive oxygen species (ROS) production and mitochondrial permeability transition pore (mPTP) activation, ultimately reducing myocardial ischemia/reperfusion injury (42–44). Another benefit of exercise on cardiovascular health includes inducing a long-term anti-inflammatory effect on the body. This is inversely related to the increased inflammation typically seen in cardiovascular disease. Myokines, which are released from skeletal muscle during physical activity, play a pivotal role in mediating these anti-inflammatory effects and promote inter-tissue communication to mediate further cardiovascular benefits. Physical activity enhances myocardial perfusion and elevates high-density lipoprotein (HDL) cholesterol levels, collectively alleviating strain on the heart and enhancing cardiovascular function in both healthy individuals and those with underlying conditions (45–48).

It is important to underscore that aerobic exercise, in addition to major effects on cardiovascular health, also has a significant impact on the neurovascular system and brain function in both physiological and pathological conditions. Many clinical and pre-clinical studies demonstrate substantial structural and functional changes in the brain induced by aerobic exercise. Specifically, moderate to high-intensity aerobic exercise promotes neurogenesis and enhances synaptic plasticity, driven by elevated release of neurotrophic growth factors, such as brain-derived neurotrophic factor (BDNF), insulin-like growth factor-I (IGF-I), vascular endothelial growth factor (VEGF), and nerve growth factor (NGF) (49–52). Studies with animals and humans have shown that aerobic exercise over extended periods enhances brain activity and increases the size of various brain regions, such as the prefrontal, parietal, and temporal cortices (53–55). Furthermore, several clinical studies demonstrate the effects of aerobic exercise on improving cerebral blood flow, promoting neuroplasticity, and increasing whole-body VO2 peak oxygen consumption in patients with stroke (56–60). Additionally, engaging in high-intensity exercise has been shown to increase brain glutamate and gamma-amino-butyric acid (GABA) levels (61, 62).

The changes induced by aerobic exercise play a crucial role in post-stroke neurovascular health as they directly contribute to neural stem cell differentiation, neuronal plasticity, and have neuroprotective effects. For example, it was shown that increased VEGF levels are central to stimulating angiogenesis around the lesion and facilitating neurological recovery post-acute stroke (63, 64). Similarly, aerobic exercise significantly increases levels of BDNF and its primary receptor, tropomyosin receptor kinase B (TrkB), involved in neuroprotective effects during conditions like cerebral ischemia and neurotoxicity, as well as synaptotagmin, a synaptic protein crucial for learning and memory (65–69). Importantly, clinical studies have demonstrated the impact of aerobic exercise training on cortical excitability and alterations in neural circuits in individuals with stroke (70–74). Preclinical studies have unveiled additional mechanistic insights into the effects of aerobic exercise, including regulation of neuro-inflammation and mitochondrial biogenesis, as well as the repair of the blood-brain barrier (75–77). Overall, the current body of evidence strongly indicates that aerobic exercise is associated with changes in brain function and can potentiate neuroplasticity, which is vital following stroke.

### Structure of cardiac rehabilitation

The effects of CR, of which aerobic exercise is an integral component are multifaceted with numerous studies, both clinical and pre-clinical, demonstrating its multi-system benefits. CR is a well-established rehabilitation treatment for individuals recovering from cardiac disease. CR has evolved from exercise only to a comprehensive treatment program that includes: physician-prescribed exercise; cardiac risk factor modification, including behavioral and lifestyle health education (nutrition, smoking cessation, as well as lipid and blood pressure management), psychosocial counseling, behavioral intervention, outcomes assessment and physician supervision (78, 79). The specific exercise prescription includes intensity (dose), frequency, duration, and progression. CR traditionally consists of three phases, with the first two delivered in a supervised hospital/center-based setting (80). Phase I refers to inpatient rehabilitation during the index hospitalization. Phase II refers to physician-supervised, outpatient-monitored physical activity several months after discharge. Individuals participate in up to 36 sessions in a graduated exercise program. Phase III consists of continuing to an unmonitored outpatient exercise program. The Centers for Medicare & Medicaid Services (CMS) has determined that the evidence is sufficient to support that cardiac rehabilitation is reasonable and necessary following acute myocardial infarction within the preceding 12 months, coronary artery bypass graft (CABG), stable angina pectoris, heart valve repair or replacement, percutaneous transluminal coronary angioplasty (PTCA) or coronary stenting, heart or heart-lung transplant and stable chronic systolic heart failure (79). Multimodal rehabilitative interventions including exercise training, lifestyle modification, and psychological intervention, has proven to be an effective strategy to improve cardiovascular health and overall well-being after cardiovascular disease (81).

### Benefits of cardiac rehabilitation for patients with cardiac and vascular disease

The numerous benefits of CR after cardiac disease led to the adoption of recommendations for CR as standard of care after cardiovascular disease. Recent systematic review and meta-analysis of exercise-based CR in people with existing coronary heart disease shows a reduction in cardiovascular mortality (relative risk: 0.74; 95% confidence interval: 0.64–0.86) and a reduction in the risk of hospital admissions (relative risk: 0.82; 95% confidence interval: 0.70–0.96). Most studies (70%) showed higher levels of health-related quality of life in 1 or more domains following exercise-based CR compared with control subjects (80). Another systematic review and meta-analysis showed that CR reduces cardiovascular mortality, recurrent cardiac events, hospitalizations and improves health-related quality of life, highlighting the cost-effectiveness of CR (82). As such, the American Heart Association (AHA) and American College of Cardiology (ACC) consider CR a Class I indication for several cardiac conditions (83). Similarly, for patients with peripheral artery disease (84), the AHA/ACC has given Class I, Level A support for a supervised exercise program similar to cardiac rehabilitation. Based on the strength of existing evidence-based research, clinical practice guidelines approve cardiac rehabilitation as an effective adjunct to medical management to improve outcomes in patients after cardiac and vascular disease.

### Benefits of cardiac rehabilitation for patients with stroke

**Figure 2:**
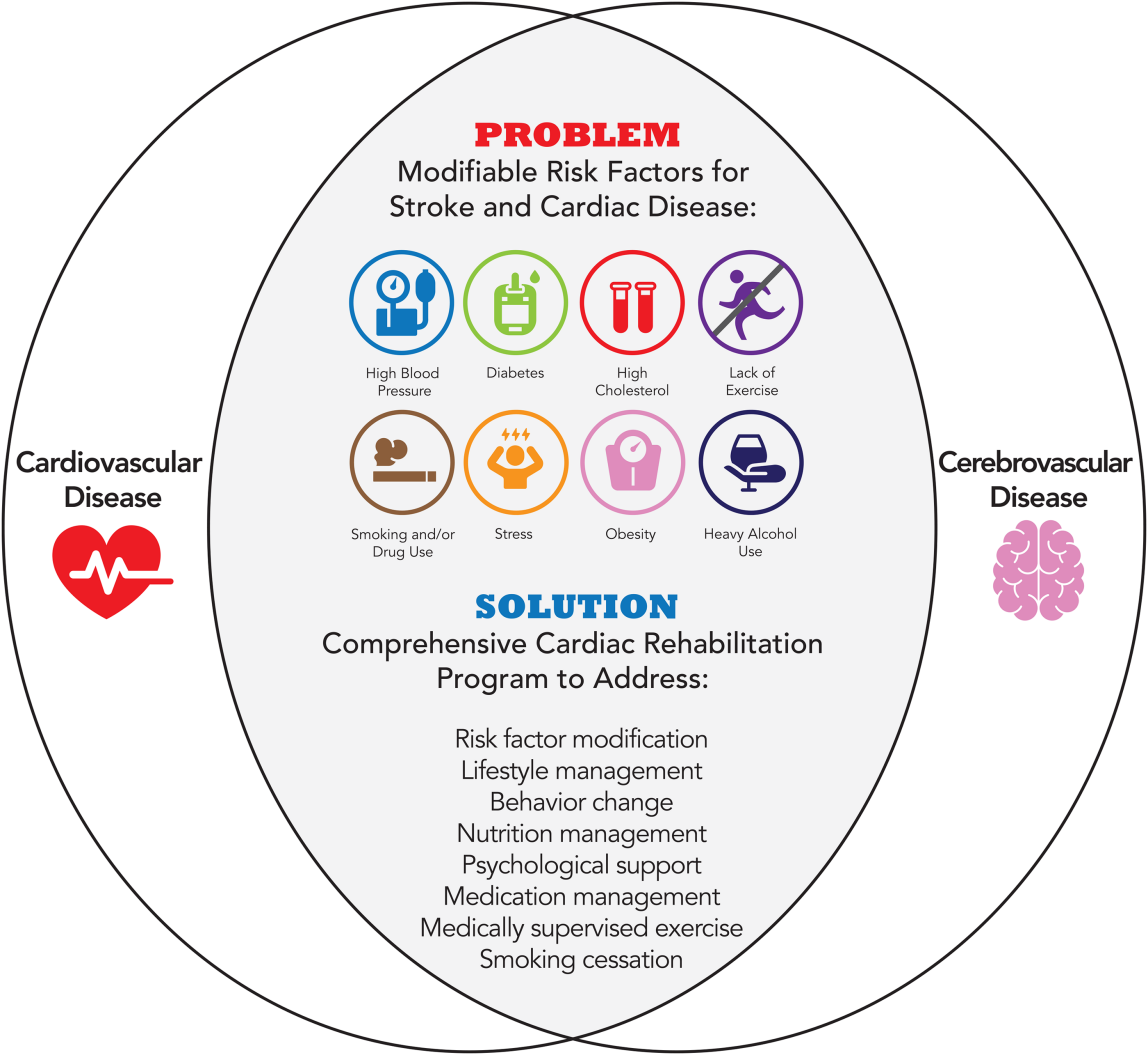
Modifiable risk factors for cardiovascular and cerebrovascular disease addressed by cardiac rehabilitation.

In the United States, stroke survivors benefit from aerobic programs with similar dosing to cardiac rehabilitation. Individuals with stroke have many of the same risk factors as individuals with cardiac disease (Figure 2). Current practice guidelines after stroke recommend structured aerobic exercise for at least 3–5 days per week, for a minimum of 20 min per session, with at least 5 min warm-up and cool-down periods (15, 85, 86). Preliminary evidence demonstrates that high-intensity interval training is associated with improvements in functional, cardiovascular, and neuroplastic outcomes post-stroke (87). Exercise programs that include aerobic exercise can improve aerobic capacity, walking ability, vascular health, and quality of life of stroke survivors. Specifically, exercise programs comprised of moderate intensity, 3 days per week, for 20 weeks should be considered for greater effect on cardiorespiratory fitness, muscle strength, and walking capacity in stroke patients (88). Exercise is also able to positively affect cognitive performance and neurovascular health in patients with known vascular disease, with a potential dose-response relationship (89). Beyond aerobic exercise alone, studies have acknowledged the feasibility and effectiveness of adapting exercise-based cardiac rehabilitation interventions for individuals with stroke who have mild to moderate disability (32, 33, 90, 91). Furthermore, comprehensive models like CR that integrate exercise, lifestyle modification, and medication management are also beneficial for individuals after TIA or stroke (18, 34, 92). Stroke survivors who participated in a comprehensive stroke recovery program incorporating modified cardiac rehabilitation had decreased all-cause mortality, improved overall function, improved cardiovascular performance (31, 93), and showed a 22% reduction in acute care hospital readmissions (94). Comprehensive programs that show clinical promise include secondary prevention strategies that integrate exercise interventions into a comprehensive risk-reduction program for stroke survivors (15, 95, 96).

In addition to recommendations that all eligible stroke survivors receive an inpatient rehabilitation stay for comprehensive interprofessional post-stroke care (Class 1, Level B) (16), in patients with deficits after stroke that impair their ability to exercise, supervision of an exercise program by a health care professional such as a physical therapist or cardiac rehabilitation professional, can be beneficial for secondary stroke prevention (Class 2a, Level-Expert Opinion) (29).

### Benefits of physical activity on cardiovascular health and mortality

Several of the benefits of CR are a result of supervised and progressive exercise training to promote sustained physical activity. In 2010, the American Heart Association defined a novel construct of cardiovascular health to promote a paradigm shift from a focus solely on disease treatment to one inclusive of positive health promotion and preservation across the life course in populations and individuals (97). More recently, the elements of this construct were updated, and the American Heart Association issued a presidential advisory introducing an enhanced approach to assessing cardiovascular health: Life's Essential 8. The components of Life's Essential 8 include diet, physical activity, nicotine exposure, sleep health, body mass index, blood lipids, blood glucose, and blood pressure (98). Of these components, physical activity appears to have an effect on all the other components. Interventions that include physical activity have a greater effect on adherence to recommended diets than interventions that do not include physical activity, including in individuals with obesity (99–101). Aerobic exercise can assist with smoking cessation (102), and improve sleep health (103–105). Supervised aerobic exercise training was effective in reducing fasting plasma glucose (9.38 mg/dl lower), total cholesterol (20.24 mg/dl lower), triacylglycerol (19.34 mg/dl lower), and low-density lipoprotein cholesterol (11.88 mg/dl lower) (106, 107). Aerobic exercise reduces blood pressure in both hypertensive and normotensive persons (108). Exercise training increases VO_2max_, along with cardiovascular capacity and endurance (78). In addition. exercise training has multiple other beneficial effects including improving endothelial function, and cardiac mitochondrial function (78, 109, 110). Thus, the promotion of a healthy lifestyle including aerobic exercise as prescribed in cardiac rehabilitation plays a critical role in optimizing cardiometabolic health in patients with vascular diseases (e.g., cardiovascular disease, cerebrovascular disease, and peripheral vascular disease) (111).

The benefits of aerobic activity extend beyond the vascular system. In addition to cerebrovascular and cardiovascular diseases (e.g., stroke, coronary artery disease, chronic heart failure, and peripheral vascular disease), aerobic activity is recommended for the treatment of various conditions including chronic kidney disease, Parkinson's disease, Alzheimer's disease, chronic obstructive pulmonary disease, low back pain, osteoporosis, osteoarthritis, obesity, depression, anxiety disorders, and several cancers (e.g., colon cancer, prostate cancer, lung cancer) (112). In a study of 750,302 U.S. veterans aged 30–95 years, cardiorespiratory fitness was measured using peak METs achieved during a standardized exercise treadmill test, and they were followed for a median of 10.2 years. Cardiorespiratory fitness was inversely associated with all-cause mortality and graded across the age spectrum, sex, and race. The mortality risk for the least fit individuals (20th percentile) was 4-fold higher compared with extremely fit individuals, and being unfit carried a greater risk than any of the other risk factors examined, including smoking, diabetes, cardiovascular disease, and hypertension (113). These data suggest that it is imperative to facilitate physical fitness through aerobic exercise, particularly in individuals who are at increased risk for cardiovascular and cerebrovascular disease.

### Future directions and recommendations

Despite published guidelines and robust evidence on the benefits of aerobic exercise for cardiovascular and cerebrovascular health, the majority of the US population does not meet current recommendations. It is therefore critical to effectively motivate and initiate behavior change, especially in clinical populations. A recent science advisory from the American Heart Association presented a framework, the 5A Model (assess, advise, agree, assist, and arrange) and strategies to promote efficient lifestyle-related behavior change counseling for patients with cardiovascular disease (114).

For patients with stroke, these arrangements should ideally include CR which has been found to be safe and effective in this population. However, presently, stroke survivors are excluded from standard cardiovascular conditioning programs as part of the standard of care. There is mounting evidence showing that exercise therapies or other rehabilitation strategies delivered during the early stages of recovery post-stroke (subacute phase; 1 week to 6 months) amplify spontaneous recovery and enhance the biological recovery process (115–117). Decreased cardiovascular capacity is one of the main reasons for limited activity and a major contributor to excess morbidity, hospitalization, mortality, and poor quality of life post-stroke. Currently, 50%–60% of stroke patients are readmitted to the hospital within the first year post their stroke event (118, 119). Hence, it is critical to prioritize improvement in cardiovascular health in the stroke survivor population.

A limitation of this narrative review is that it does not provide a quantitative assessment of the effects of CR for stroke survivors. Future meta-analysis to evaluate the benefits of CR may compare the timing of the initiation of a CR program, the aerobic exercise prescription (dose, duration, and intensity), as well as type of stroke.

Structured, medically supervised programs like CR, which address physical activity, aerobic exercise, risk factor education, and behavior change, definitively improve the overall health of cardiac patients (80, 120) and reduce hospital readmissions, secondary cardiovascular events, and mortality (121, 122). Since stroke survivors have many of the same risk factors as cardiac patients (e.g., hypertension, diabetes, hyperlipidemia, and obesity), a comprehensive program such as CR could potentially improve their quality of life and vascular health, as well as reduce hospital readmission and mortality (31, 94). Future studies should demonstrate the value of adding cardiac rehabilitation to standard neurorehabilitation in reducing disability, and all-cause mortality for individuals with stroke.
